# Decreased renal function and associated factors in cities, towns and rural areas of Tanzania: a community‐based population survey

**DOI:** 10.1111/tmi.12651

**Published:** 2015-12-28

**Authors:** Robert Peck, Kathy Baisley, Bazil Kavishe, Jackson Were, Janneth Mghamba, Liam Smeeth, Heiner Grosskurth, Saidi Kapiga

**Affiliations:** ^1^ Mwanza Intervention Trials UnitMwanzaTanzania; ^2^ Weill Bugando School of MedicineMwanzaTanzania; ^3^ London School of Hygiene & Tropical MedicineLondonUK; ^4^ MRC Uganda Research Unit on AIDSEntebbeUganda; ^5^ Tanzanian Ministry of Health and Social WelfareDar es SalaamTanzania

**Keywords:** renal disease, Tanzania, epidemiology, prevalence, risk factors, adults, maladie rénale, Tanzanie, épidémiologie, prévalence, facteur de risque, adultes

## Abstract

**Objectives:**

Data on renal dysfunction in sub‐Saharan Africa, comparing urban and rural areas, have not yet been reported. Therefore, we aimed to determine the distribution of low estimated glomerular filtration rates (eGFRs) in urban and rural Tanzania, to describe factors associated with low eGFR and to quantify fractions attributable to common risk factors.

**Methods:**

We conducted a community‐based survey of 1095 randomly selected Tanzanian adults (≥18 years). A structured questionnaire and examinations were used to document sociodemographic characteristics, diet, physical activity, anthropomorphic measurements and blood pressure. Blood tests were performed for HIV infection, diabetes mellitus and creatinine. eGFR was calculated using two equations recommended for African adults.

**Results:**

Serum creatinine was available for 1043 participants: 170 in Mwanza city, 326 in district towns and 547 in rural areas. Mean age was 35.5 years and 54% were females. The prevalence of eGFR < 60 ml/min/1.73 m^2^ in these 3 strata was 2.3% (95% CI = 0.8–6.6%), 7.5% (4.7–11.8%) and 7.4% (5.1–10.6%), respectively. When age standardised to the WHO world population, prevalences were 3.8%, 10.1% and 8.1%. Factors associated with low eGFR included district town residence, older age, greater wealth, less physical activity and hypertension. Only 21% of cases with eGFR < 60 ml/min/1.73 m^2^ were attributable to HIV, hypertension or diabetes.

**Conclusions:**

Decreased renal function is common in Tanzania, particularly in district towns, and unique risk factors for kidney disease may exist in this population. Population‐specific strategies for prevention, early diagnosis and treatment of kidney disease are needed for Africa.

## Abstract (fr)

**Objectifs:**

Les données sur la dysfonction rénale en Afrique subsaharienne, comparant les zones urbaines et rurales, n'ont pas encore été rapportées. Par conséquent, nous avons cherché à déterminer la distribution des taux estimés de filtration glomérulaire (eGFR) faibles en zone urbaine et rurale de la Tanzanie; décrire les facteurs associés à au eGFR faible et quantifier les fractions attribuables à des facteurs de risque courants.

**Méthodes:**

Nous avons mené une enquête communautaire sur 1095 adultes (≥ 18 ans) tanzaniens choisis au hasard. Un questionnaire structuré et des examens ont été utilisés pour documenter les caractéristiques sociodémographiques, l'alimentation, l'activité physique, les mesures anthropomorphiques et la pression artérielle. Des tests sanguins ont été effectués pour l'infection à VIH, le diabète sucré et la créatinine. eGFR a été calculé en utilisant 2 équations recommandées pour les adultes africains.

**Résultats:**

La créatinine sérique était disponible pour 1043 participants: 170 dans la ville de Mwanza, 326 dans les villes de district et 547 dans les zones rurales. L’âge moyen était de 35,5 ans et 54% étaient des femmes. La prévalence du eGFR <60 ml/min/1,73m^2^ dans ces 3 strates était de 2,3% (IC95% = 0,8 à 6,6%), 7,5% (4,7 à 11,8%) et 7,4% (5,1 à 10,6%) respectivement. Lorsqu'ajustées selon l’âge pour la population mondiale de l’OMS, ces prévalences étaient de 3,8%, 10,1% et 8,1%. Les facteurs de risque associés à un faible eGFR comprenaient la résidence dans un quartier de ville, l’âge plus élevé, une plus grande richesse, moins d'activité physique et l'hypertension. Seuls 21% des cas avec eGFR <60 ml/min/1,73m^2^ étaient attribuables au VIH, à l'hypertension ou au diabète.

**Conclusions:**

La diminution de la fonction rénale est fréquente en Tanzanie, en particulier dans les villes de district et des facteurs de risque uniques pour la maladie rénale existeraient dans cette population. Des stratégies de prévention spécifiques à la population, le diagnostic précoce et le traitement de la maladie rénale sont nécessaires en Afrique.

## Abstract (es)

**Objetivos:**

Aún no se han reportado datos sobre disfunción renal en África subsahariana, comparando áreas urbanas y rurales. Por lo tanto, nuestro objetivo era determinar la distribución de las tasas de filtración glomerular estimada (TFGe) bajas en zonas urbanas y rurales de Tanzania; describir los factores asociados con TFGe bajas y cuantificar las fracciones atribuibles a factores de riesgo comunes.

**Métodos:**

Hemos realizado un estudio basado en la comunidad a 1095 adultos tanzanos elegidos al azar (≥18 años). Se utilizaron un cuestionario estructurado y exámenes para documentar las características socio demográficas, dieta, actividad física, medidas antropomórficas y presión sanguínea. Se realizaron pruebas en sangre para determinar la infección por VIH, diabetes mellitus y creatinina. Se calculó la TFGe utilizando 2 ecuaciones recomendadas para adultos africanos.

**Resultados:**

La creatinina en suero estaba disponible para 1043 participantes: 170 en la ciudad de Mwanza, 326 en ciudades distritales y 547 en áreas rurales. La edad media era de 35.5 años y un 54% eran mujeres. La prevalencia de TFGe <60mL/min/1.73m^2^ en estos 3 estratos era del 2.3% (IC 95% = 0.8–6.6%), 7.5% (4.7–11.8%) y 7.4% (5.1–10.6%) respectivamente. Al estandarizar por edad frente a la población estándar mundial de la OMS, las prevalencias eran 3.8%, 10.1% y 8.1%. Los factores asociados con una TFGe baja incluían residir en ciudades distritales, mayor edad, mayor riqueza, menor actividad física e hipertensión. Solo un 21% de los casos con TFGe<60mL/min/1.73m^2^ podían atribuirse al VIH, la hipertensión o la diabetes.

**Conclusiones:**

La insuficiencia renal es común en Tanzania, particularmente en las ciudades distritales y podrían existir factores de riesgo únicos para enfermedad renal entre esta población. En África se requieren estrategias específicas según la población para la prevención, diagnóstico temprano y tratamiento de la enfermedad renal.

## Introduction

Despite the rising tide of non‐communicable disease (NCD) in sub‐Saharan Africa (SSA), few studies have assessed the burden of kidney disease in this region 1. Health facility data indicate that kidney disease is common, including our own study from western Tanzania, which demonstrated that kidney disease was the 6th leading cause of hospital deaths 2. Studies of the general population, though, are lacking. According to a recent meta‐analysis, only three high‐quality epidemiological surveys had been conducted in SSA, and none had compared the prevalence of kidney disease in urban and rural settings 1.

Risk factors for kidney disease in SSA are also poorly described and may differ from those in the United States and Europe 3, 4, and between urban and rural areas 3, 5. Infectious risk factors for kidney disease remain common in SSA, particularly in rural areas 5. Non‐infectious risk factors such as diabetes, hypertension and obesity are now becoming increasingly prevalent, particularly in urban areas 6. Better understanding of risk factors for kidney disease in sub‐Saharan Africa is critical for planning clinical trials and improving services 3, 4, 5.

We determined estimated glomerular filtration rates (eGFRs) in a large community survey of randomly selected, representative samples of adults in rural communities, district towns and urban areas in north‐western Tanzania. The objectives of our study were as follows: (i) to determine the distribution of decreased eGFR in the three study locations, (ii) to systematically describe factors associated with low eGFR and (iii) to quantify the proportion of low eGFRs that were attributable to HIV, hypertension and diabetes.

## Methods

### Study design and sampling

This study was part of a larger population survey of chronic diseases that we conducted between May 2012 and April 2013 among adults (≥18 years) in north‐western Tanzania (in the Lake Zone located just south of Lake Victoria). We used stratified, multistage sampling, with five strata: a municipal area (Mwanza city), two district towns (Geita and Kahama) and the rural communities corresponding to each district town. The adults population of these municipal areas, district towns and rural communities are similar to other parts of East Africa with adult median age of ~33 years, ~55% females, a median income of ~40 USD, ~25% secondary education or higher and ~6% prevalence of HIV 7. The major tribe in the study area is the Sukuma tribe, a Bantu ethnic group. We took an independent two‐stage self‐weighting sample from each stratum, firstly sampling areas with probability proportional to the number of households, and secondly randomly sampling households within these areas. Households (the primary sampling unit) were eligible if they were located within 5 km of a health facility. Selected households were visited, verbal consent from household heads was obtained, a list of adult household members prepared, and all resident adults were invited to participate. Consenting participants were recruited. The sampling strategy is described in greater detail elsewhere 7.

### Data collection

Consenting participants were interviewed in their homes or communal places using a structured questionnaire adapted from the WHO STEPwise approach to chronic disease risk factor surveillance (STEPS) instrument 8. We collected information on sociodemographic characteristics and risk factors for non‐communicable diseases (NCDs) including alcohol use, smoking, diet and physical activity. Weight and height were measured according to the STEPS protocol. Three blood pressure (BP) measurements were taken in seated participants after resting for at least 15 min, using Omron^®^ digital automatic BP monitor model M6 (Omron Health Care Manufacturing Vietnam Co., Ltd, Binh Duong Province, Vietnam). The third reading was used for analysis.

### Laboratory methods

At the time of enrolment, venous blood samples were obtained. Serum aliquots were stored in a portable freezer (−20 °C) and transported (within 5 days) to the National Institute for Medical Research Laboratory in Mwanza city, where they were stored at −80 °C. Serum samples were later transported to the MRC/UVRI Uganda Research Unit on AIDS in Entebbe, where serum creatinine was measured using Cobas Integra 400 Plus Analyzer (Roche Diagnostic Limited, Switzerland), calibrated by the creatinine Jaffe 2 method. Capillary random blood glucose (RBG) was measured using a portable, battery‐driven Accu‐Check^®^ Aviva (Roche Diagnostics GmbH, Mannheim, Germany), and rapid HIV diagnostic tests were performed according to national protocols. In study subjects with an elevated RBG (≥7 mmol/l), a fasting blood sample was subsequently obtained for measurement of fasting blood glucose (FBG). Other laboratory methods were previously described in detail 7.

### Definitions

The primary outcome of this study was decreased renal function according to the estimated glomerular filtration rate (eGFR). For this study, decreased renal function was defined as eGFR<60 ml/min/1.73 m^2^ according to the Kidney Disease Improving Global Outcomes (KDIGO) guidelines 9. The eGFR was calculated using the CKD‐EPI equation as this is recommended by KDIGO and is the most accurate eGFR equation for African adults 9, 10, 11, 12. The grade of renal function was also defined according to KDIGO guidelines: normal (eGFR≥90); mildly decreased (eGFR 60–89); mildly–moderately decreased (eGFR 45–59); moderately–severely decreased (eGFR 30–44); severely decreased (eGFR 15–29); and kidney failure (eGFR < 15) 9. For the sake of comparison, we also calculated the eGFR using the Modified Diet Renal Disease (MDRD) equation. All eGFR calculations were performed without the coefficient for ethnicity because this coefficient has been shown to overestimate the actual glomerular filtration rate among Africans 10, 11, 12.

Hypertension was defined as systolic blood pressure ≥140 mmHg and/or diastolic blood pressure ≥90 mmHg or current antihypertensive therapy 13. Diabetes mellitus was defined as FBG ≥7 mmol/L or RBG >11.1 mmol/l or being on diabetes medication. Body mass index (BMI, kg/m2) was classified as underweight (<18.5), normal (18.5 to <25), overweight (25 to <30) and obese (≥30). Waist circumference >94 cm and >80 cm was classified as above normal (central obesity) for males and females.

### Statistical analysis

Data were double entered and verified in OpenClinica^®^ version 3.0.1 (OpenClinica, Waltham, USA). We used the survey commands of Stata version 13 (Stata Corporation, College Station, USA) to account for the complex sampling design, with standard errors adjusted for clustering at the level of the primary sampling unit. We applied strata sampling weights to account for differential probability of selection between strata, as has been described in greater detail elsewhere 7.

We estimated the population prevalence of decreased renal function based on the CKD‐EPI and MDRD equations, stratified by location (Mwanza city, district towns and rural), using weighted percentages and confidence intervals (CI) but observed (i.e. unweighted) frequencies. In addition, prevalences were age standardised using both the United States 2000 population and the WHO world population as references as we have previously reported for hypertension and diabetes mellitus 7.

We assessed the agreement of the classification based on the MDRD with that based on the CKD‐EPI equation using a Kappa statistic, with the Landis and Koch interpretation for the strength of agreement 14.

We used logistic and linear regression to investigate factors associated with the prevalence of decreased renal function (eGFR < 60 ml/min/1.73 m^2^, based on CKD‐EPI equation) and of mean eGFR, respectively, and estimated regression coefficients (odds ratios (OR) and mean change) and 95% CI using the Stata survey procedures to adjust the standard errors for the survey design. Sampling weights were not applied in the risk factor analyses. We adjusted for age, sex and location *a priori* in all models, so comparisons were essentially within location. We estimated the population attributable fraction (PAF) of decreased renal function for HIV, hypertension and diabetes using the adjusted ORs from the final model. The joint PAF for HIV, hypertension and diabetes was calculated by generating a new variable to represent the eight possible combinations of the 3 CDs: none of the 3, HIV only, hypertension only, diabetes only, HIV and hypertension, HIV and diabetes, hypertension and diabetes, and all 3 CDs. This new variable was then included in a logistic regression model adjusted for age, sex and location. The adjusted (a)ORs from this model multiplied by the proportion of cases of low eGFR in each of the categories by the attributable fraction in the exposed (1‐aOR)/aOR); these values were then summed to get the overall PAF.

Potential determinants of mean eGFRs were examined using a conceptual framework with three levels. Age, sex and location were included in all models. Sociodemographic factors (e.g. education, marital status, income group) were added one by one to the age‐, sex‐ and location‐adjusted analysis and retained if associated with mean eGFR at *P* < 0.10. Behavioural factors (e.g. smoking, diet, alcohol intake) were then added one by one and retained if they remained associated at *P* < 0.10. Associations with clinical factors (e.g. body mass index, hypertension, diabetes) were subsequently determined in a similar way. This strategy allowed us to assess the effects of variables at each level of the framework, adjusted for more distal variables.

### Ethical considerations

This study was approved by the ethics committees of the Tanzania National Institute for Medical Research and the London School of Hygiene and Tropical Medicine. We obtained written informed consent from all participants (or by literate witnesses for illiterate participants) before administering study procedures. Participants were interviewed in private to ensure confidentiality. Participants with any untreated or newly diagnosed conditions referred to a local health facility for further assessment, counselling, and long‐term care and treatment. All procedures were consistent with ethical standards of the Helsinki Declaration.

## Results

### Enrolment

Between August 2012 and April 2013, we sampled 625 households of which 563 (90%) agreed to participate. There were 1374 eligible adults in the participating households, and 1095 (80% of those eligible) consented to the survey, resulting in an estimated 72% response rate. Among adults surveyed, creatinine measurements were available for 1043: 170 of 175 (97%) adults in Mwanza city, 326 of 344 (95%) in district towns and 547 of 576 (95%) in rural areas.

### Characteristics of the study population

Among 1043 study participants with data on eGFR, the mean age was 35.5 years (SD = 15.3 years) and 566 (54.3%) were female; 852 (81.7%) had only a primary education or less and 731 (74.4%) earned <64 USD per month (Table 1). Regarding clinical characteristics, 197 (18.5%) were overweight or obese, 180 (17.3%) had hypertension, 9 (0.9%) had diabetes mellitus and 87 (8.4%) were HIV seropositive.

**Table 1:** Population characteristics and factors associated with decreased renal function (eGFR <60 ml/min/1.73 m^2^) among adults in a population survey in north‐western Tanzania, 2012–2013

	Total N (column %)^a^	Prevalence of eGFR < 60 (row %)^a^	Unadjusted OR (95% CI)^b^	Age‐, sex‐, and residence‐adjusted OR (95% CI)^b^
Sociodemographic				
Residence			*P* = 0.11	*P* = 0.12
Mwanza city	170 (16.3%)	4 (2.4%)	1	1
District towns	326 (31.3%)	23 (7.1%)	3.15 (0.97–10.27)	3.37 (1.07–10.64)
Rural	547 (52.4%)	42 (7.7%)	3.45 (1.09–10.89)	2.96 (0.94–9.33)
Age group			*P* < 0.001	*P* < 0.001
<25 years	293 (28.1%)	10 (3.4%)	1	1
25–34 years	326 (31.3%)	16 (4.9%)	1.46 (0.68–3.15)	1.45 (0.68–3.08)
35–44 years	167 (16.0%)	12 (7.2%)	2.19 (1.27–3.77)	2.16 (1.27–3.66)
≥45 years	257 (24.6%)	31 (12.1%)	3.88 (2.18–6.90)	3.82 (2.16–6.74)
Sex			*P* = 0.34	*P* = 0.52
Male	477 (45.7%)	35 (7.3%)	1	1
Female	566 (54.3%)	34 (6.0%)	0.81 (0.52–1.26)	0.87 (0.57–1.34)
Education			*P* = 0.12	*P* = 0.76
Secondary or above	191 (18.3%)	9 (4.7%)	1	1
Completed primary	491 (47.1%)	29 (5.9%)	1.27 (0.55–2.94)	0.91 (0.38–2.20)
None/incomplete primary	361 (34.6%)	31 (8.6%)	1.90 (0.90–4.03)	1.12 (0.47–2.66)
Marital status			*P* = 0.06	*P* = 0.49
Married/living as married	674 (64.6%)	45 (6.7%)	1	1
Divorced/sep/widowed	163 (15.6%)	17 (10.4%)	1.63 (0.90–2.94)	1.46 (0.78–2.71)
Single	206 (19.8%)	7 (3.4%)	0.49 (0.22–1.12)	0.85 (0.24–3.03)
Income per month^c^			*P* = 0.30	*P* = 0.18
Upper tertile (64 + USD)	251 (25.6%)	13 (5.2%)	1	1
Middle tertile (26–63 USD)	262 (26.7%)	22 (8.4%)	1.68 (0.86–3.28)	1.87 (0.95–3.69)
Lower tertile (<26 USD)	469 (47.8%)	30 (6.4%)	1.25 (0.66–2.36)	1.29 (0.65–2.58)
Items owned			*P* = 0.02	*P* = 0.02
5+	262 (25.1%)	24 (9.2%)	1	1
2–4	668 (64.0%)	35 (5.2%)	0.55 (0.36–0.84)	0.54 (0.35–0.83)
0–1	113 (10.8%)	10 (8.8%)	0.96 (0.43–2.15)	0.80 (0.33–1.91)
Behavioural				
Smoking			*P* = 0.18	*P* = 0.89
Never smoked	856 (82.1%)	52 (6.1%)	1	1
Ex–smoker	79 (7.6%)	8 (10.1%)	1.74 (0.91–3.35)	1.17 (0.60–2.29)
Current smoker	108 (10.4%)	9 (8.3%)	1.41 (0.62–3.19)	0.94 (0.38–2.31)
Alcohol consumption			*P* = 0.34	*P* = 0.57
Never drinks	669 (64.1%)	40 (6.0%)	1	1
None in past 12 months	237 (22.7%)	21 (8.9%)	1.53 (0.84–2.80)	1.15 (0.60–2.23)
Drinking in past 12 months	137 (13.1%)	8 (5.8%)	0.98 (0.38–2.52)	0.71 (0.28–1.80)
Eats fruit/veg less than 5 days/week			*P* = 0.05	*P* = 0.11
No	771 (73.9%)	58 (7.5%)	1	1
Yes	272 (26.1%)	11 (4.0%)	0.52 (0.27–1.00)	0.58 (0.30–1.14)
Days of vigorous physical activity/week^d^			*P* = 0.26	*P* = 0.07
5+	500 (48.0%)	28 (5.6%)	1	1
1–4	135 (13.0%)	8 (5.9%)	1.06 (0.50–2.27)	1.31 (0.60–2.85)
None	407 (39.1%)	33 (8.1%)	1.49 (0.88–2.52)	1.81 (1.09–3.00)
Clinical				
Body Mass Index (BMI) category (kg/m^2^)^e^			*P* = 0.61	*P* = 0.83
Underweight (<18.5)	109 (10.5%)	11 (10.1%)	1.73 (0.69–4.29)	1.52 (0.62–3.77)
Normal (18.5–<25)	737 (71.0%)	45 (6.1%)	1	1
Overweight (25–<30)	123 (11.8%)	8 (6.5%)	1.07 (0.57–2.02)	0.97 (0.49–1.91)
Obese (≥30)	69 (6.6%)	5 (7.2%)	1.20 (0.51–2.81)	0.92 (0.37–2.28)
Central Obesity^f^			*P* = 0.97	*P* = 0.70
No	784 (75.2%)	52 (6.6%)	1	1
Yes	259 (24.8%)	17 (6.6%)	0.99 (0.52–1.90)	0.85 (0.38–1.94)
Hypertension			*P* = 0.003	*P* = 0.11
No	863 (82.7%)	46 (5.3%)	1	1
Yes	180 (17.3%)	23 (12.8%)	2.60 (1.41–4.78)	1.84 (0.87–3.89)
Systolic blood pressure (mmHg)				
<120	448 (43.0%)	23 (5.1%)	*P* = 0.005 1.20 (1.06–1.36)^i^	*P* = 0.14 1.12 (0.96–1.30)^i^
120–140	445 (42.7%)	28 (6.3%)		
>140	150 (14.4%)	18 (12.0%)		
Diastolic blood pressure (mmHg)				
<80	748 (71.7%)	40 (5.3%)	*P* = 0.008 1.35 (1.09–1.68)^h^	*P* = 0.13 1.22 (0.94–1.57)^h^
80–90	210 (20.1%)	16 (7.6%)		
>90	85 (8.1%)	13 (15.3%)		
Diabetes^g^			*P* = 0.54	*P* = 0.84
No	1031 (99.1%)	67 (6.5%)	1	1
Yes	9 (0.9%)	1 (11.1%)	1.80 (0.26–12.24)	1.21 (0.19–7.84)
HIV positive^h^			*P* = 0.06	*P* = 0.08
No	947 (91.6%)	59 (6.2%)	1	1
Yes	87 (8.4%)	9 (10.3%)	1.74 (0.99–3.06)	1.68 (0.93–3.04)

a Actual number of respondents and proportions, without sampling weights applied.

b Standard errors and 95% CI adjusted for clustering in survey design.

c Missing for 61 participants.

d Missing for one participant. Vigorous physical activity was defined as ‘activity that causes large increases in breathing or heart rate like carrying or lifting heavy loads, very brisk walking, digging or construction work for at least 10 min continuously’.

e Missing for five participants.

f Central obesity was defined as a waist circumference of >0.94 for women and waist circumference >0.80 for men.

g Missing for three participants.

h HIV diagnosis missing for nine participants.

i OR for 10 unit mmHg increase in blood pressure, modelled as a linear association with continuous covariate.

### Renal function outcomes

**Figure 1:**
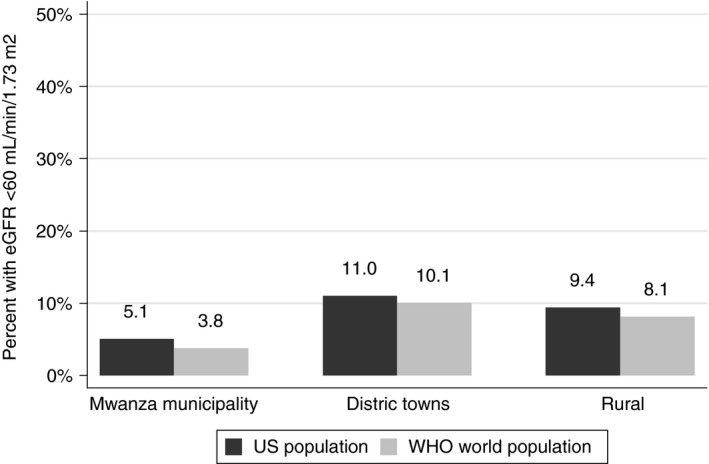
Prevalence of decreased renal function among adults ≥18 years in a population survey in north‐western Tanzania, age‐standardised to the US population and the WHO world population.

According to the CKD‐EPI equation, the mean (95% CI) eGFRs (ml/min/1.73 m^2^) were 114.5 (109.9–119.0), 110.2 (106.9–113.5) and 107.7 (105.4–110.1) in Mwanza city, district towns and rural areas, respectively (Table 2). Similar estimates were obtained with the MDRD equation. The prevalence (95% CI) of decreased renal function (defined by eGFR<60 ml/min/1.73 m^2^) was 2.3% (0.8–6.6%), 7.5% (4.7–11.8%) and 7.4% (5.1–10.6%) in Mwanza city, district towns and rural areas, respectively. There were no cases of moderately–severe or severely decreased eGFR in Mwanza city, compared with 2.7% in district towns and 2.2% in rural areas. When standardised to the US 2000 population, the prevalence of decreased renal function was 5.1%, 11.0% and 9.4% in Mwanza city, district towns and rural areas, respectively (Figure 1).

**Table 2:** Mean serum creatinine and eGFR, and prevalence of decreased renal function among adults in a population survey in north‐western Tanzania, 2012–2013

	Mwanza city (*n* = 170)	District towns (*n* = 326)	Rural (*n* = 547)
	Weighted mean (95% CI)^a^	Weighted mean (95% CI)^a^	Weighted mean (95% CI)^a^
Serum creatinine (mg/dl)	0.73 (0.69–0.77)	0.80 (0.76–0.84)	0.79 (0.76–0.81)
Estimated GFR (ml/min/1.73 m^2^)			
CKD‐EPI equation^b^,^c^	114.5 (109.9–119.0)	110.2 (106.9–113.5)	107.7 (105.4–110.1)
MDRD equation^b^,^c^	116.8 (105.9–127.7)	110.0 (105.8–114.2)	111.1 (107.1–115.0)

	Weighted % (95% CI)^a^	Unweighted N^d^	Weighted % (95% CI)^a^	Unweighted N^d^	Weighted % (95% CI)^a^	Unweighted N^d^
KDIGO Kidney disease category^e^						
Normal (eGFR ≥90)	87.4 (79.6–92.5)	150	81.6 (76.6–85.7)	266	79.4 (75.5–82.9)	432
Mildly decreased (eGFR 60–89)	10.3 (5.9–17.4)	16	10.9 (8.8–13.5)	37	13.9 (10.3–16.7)	73
Mildly–moderately decreased (eGFR 45–59)	2.3 (0.8–6.6)	4	4.8 (2.7–8.4)	15	5.3 (3.3–8.2)	30
Moderately–severely decreased (eGFR 30–44)	0	0	2.4 (0.8–6.9)	7	1.6 (0.9–2.8)	10
Severely decreased (eGFR 15–29)	0	0	0.3 (0.0–2.3)	1	0.6 (0.1–1.9)	2
Kidney failure (eGFR <15)	0	0	0	0	0	0
eGFR <60 ml/min/1.73 m^2^ (CKD‐EPI)^b^,^c^						
Yes	2.3 (0.8–6.6)	4	7.5 (4.7–11.8)	23	7.4 (5.1–10.6)	42
Age <20 years	0	0	5.6 (1.8–16.3)	2	0	0
Age 20–39 years	0	0	7.6 (4.4–12.6)	15	5.9 (3.1–10.7)	17
Age 40–59 years	8.5 (2.8–22.8)	3	5.5 (1.3–20.6)	3	9.6 (5.1–17.3)	14
Age ≥60 years	7.8 (0.8–46.2)	1	18.6 (5.6–47.1)	3	14.8 (6.9–28.7)	11
eGFR <60 ml/min/1.73 m^2^ (MDRD)^b^,^c^						
Yes	3.7 (1.5–8.5)	6	9.4 (6.0–14.5)	29	10.2 (7.8–13.2)	59

a Values are weighted estimates, adjusted for survey design with sampling weights applied.

b The CKD‐EPI eGFR calculations were used as the primary outcomes in this study. The MDRD eGFR calculation is provided for the sake of comparison.

c Without coefficient for black race.

d Actual number of respondents, without sampling weights applied.

e Based on CKD‐EPI equation without coefficient for race.

The agreement between the CKD‐EPI equation with the MDRD equations in the classification of decreased renal function (eGFR < 60 ml/min/1.73 m^2^) was excellent (kappa = 0.83) (Table 3). Study subjects with eGFR<60 ml/min/1.73 m^2^ by the CKD‐EPI equation (69/69 (100%)) also had eGFR<60 ml/min/1.73 m^2^ by the MDRD equation.

**Table 3:** Agreement between different measurements of chronic kidney disease (eGFR <60 ml/min/1.73 m^2^) using CKD‐EPI equation *vs* MDRD equation among adults in a population survey in north‐western Tanzania, 2012–2013

eGFR <60 ml/min/1.73 m^2^ (CKD‐EPI)	eGFR <60 ml/min/1.73 m^2^ (MDRD)			
	No (N, row %)^a^	Yes (N, row %)^a^	Total (N, %)^a^	Kappa statistic (MDRD *vs* CKD‐EPI)
No	949 (97.4%)	25 (2.6%)	969 (100%)	0.83
Yes	0	69 (100%)	69 (100%)	P < 0.001

a Actual number of respondents and proportions, without sampling weights applied.

### Factors associated with decreased renal function

People living in district towns had a higher odds of eGFR < 60 ml/min/1.73 m^2^ (adjusted (a)OR = 3.37, 95% CI = 1.07–10.64) (Table 1). Increasing age was strongly associated with decreased renal function. Adults aged 35–44 years and ≥45 years had 2.16 (95% CI = 1.27–3.66) and 3.82 (95% CI = 2.16–6.74) greater adjusted odds of decreased renal function compared with adults <25 years old, after adjusting for sex and residence. Similarly, people who were not engaged in vigorous physical activity had greater odds of decreased renal function (aOR = 1.81, 95% CI = 1.09–3.00), when compared with those engaged in vigorous activity ≥5 days a week. Decreased renal function was also associated with ownership of household items, with prevalence highest among those owning more items (*P* = 0.02). However, the prevalence of decreased renal function was also relatively higher among those in the lower income group *vs* the middle income group.

Hypertension was associated with decreased renal function in the unadjusted analysis (OR = 2.60, 95% CI = 1.41–4.78; *P* = 0.008) but this association was weakened in the adjusted analysis (aOR = 1.84, 95% CI = 0.87–3.89; *P* = 0.14). Similar trends were observed for both systolic and diastolic blood pressures. We also observed some evidence of an increased odds of decreased renal function among those who were HIV positive (aOR = 1.68, 95% CI = 0.93–3.04; *P* = 0.08).

### Factors associated with mean eGFR

After adjustment, people living in district towns had a lower mean eGFR (−5.89 ml/min/1.73 m^2^, 95% CI = [−10.94, −0.84]) (Table 4). Age was strongly associated with decreasing mean eGFR. After adjusting for sex and location, adults aged ≥45 years had a mean eGFR that was 35 ml/min/1.73 m^2^ lower than that of adults under 25 years of age (95% CI = [−38, −31]). In the adjusted analysis, there was also some evidence of a decrease in mean eGFR with increased ownership of household items (*P* = 0.05); however, there was no evidence of an association with other sociodemographic factors.

**Table 4:** Mean serum creatinine and eGFR, and prevalence of decreased renal function among adults in a population survey in north‐western Tanzania, 2012–2013

	Total N^a^	Mean eGFR^a^	Unadjusted regression coefficient (95% CI)^b^	Age, sex and residence‐adjusted regression coefficient (95% CI)^b^	
Sociodemographic					
Residence			*P* = 0.03	*P* = 0.08	
Mwanza city	170	114.9	0 (ref)	0 (ref)	
District towns	326	110.4	−4.58 [−10.67, 1.50]	−5.89 [−10.94, −0.84]	
Rural	547	107.2	−7.75 [−13.64, −1.87]	−4.15 [−8.92, 0.62]	
Age group			*P* < 0.001	*P* < 0.001	
<25 years	293	124.9	0 (ref)	0 (ref)	
25–34 years	326	113.8	−11.16 [−14.79, −7.53]	−11.13 [−14.69, −7.58]	
35–44 years	167	103.4	−21.53 [−26.10, −16.96]	−21.76 [−26.31, −17.21]	
≥45 years	257	90.2	−34.70 [−38.11, −31.29]	−34.87 [−38.32, −31.42]	
Sex			*P* = 0.97	*P* = 0.18	
Male	477	109.4	0 (ref)	0 (ref)	
Female	566	109.5	0.05 [−2.69, 2.79]	−1.56 [−3.87, 0.75]	
Education			*P* < 0.001	*P* = 0.48	
Secondary or above	191	116.1	0 (ref)	0 (ref)	
Complete primary	491	109.8	−6.25 [−10.44, −2.06]	1.33 [−2.65, 5.31]	
None/incomplete primary	361	105.5	−10.60 [−14.58, −6.62]	2.83 [−1.83, 7.49]	
Marital status			*P* < 0.001	*P* = 0.31	
Married/living as married	674	107.4	0 (ref)	0 (ref)	
Divorced/sep/widowed	163	99.7	−7.62 [−12.70, −2.55]	−1.53 [−5.80, 2.73]	
Single	206	124.0	16.65 [12.29, 21.02]	3.11 [−1.99, 8.21]	
Income per month			*P* = 0.64	*P* = 0.18	
Upper tertile (64 + USD)	251	108.0	0 (ref)	0 (ref)	
Middle tertile (26–63 USD)	262	109.1	1.12 [−3.71, 5.96]	0.50 [−3.89, 4.89]	
Lower tertile (<26 USD)	469	109.9	1.90 [−2.13, 5.92]	2.69 [−0.85, 6.24]	
Items owned			*P* = 0.005	*P* = 0.05	
5+	262	108.3	0 (ref)	0 (ref)	
2–4	668	111.0	2.66 [−1.80, 7.13]	3.94 [0.60, 7.27]	
0–1	113	103.0	−5.27 [−10.73, 0.20]	0.28 [−4.48, 5.04]	
Behavioural					Adjusted regression coefficient (95% CI)^b^,^c^
Smoking			*P* < 0.001	*P* = 0.41	*P* = 0.43
Never smoked	856	110.8	0 (ref)	0 (ref)	0 (ref)
Ex–smoker	79	101.1	−9.70 [−15.43, −3.97]	−1.20 [−5.51, 3.12]	−1.16 [−5.48, 3.16]
Current smoker	108	105.1	−5.69 [−11.07, −0.32]	3.35 [−1.66, 8.36]	3.16 [−1.80, 8.11]
Alcohol consumption			*P* = 0.004	*P* = 0.91	*P* = 0.82
Never drinks	669	111.4	0 (ref)	0 (ref)	0 (ref)
None in past 12 months	237	106.0	−5.40 [−9.41, −1.39]	0.16 [−3.66, 3.98]	0.20 [−3.61, 4.02]
Drinking in past 12 months	137	105.6	−5.88 [−10.55, −1.20]	0.83 [−3.08, 4.74]	1.20 [−2.67, 5.07]
Eats fruit/veg less than 5 days/week			*P* = 0.08	*P* = 0.98	*P* = 0.72
No	771	108.7	0 (ref)	0 (ref)	0 (ref)
Yes	272	111.6	2.88 [−0.37, 6.13]	0.03 [−2.80, 2.85]	−0.49 [−3.29, 2.30]
Days of vigorous physical activity/week			*P* = 0.37	*P* = 0.07	*P* = 0.09
5+	500	109.6	0 (ref)	0 (ref)	0 (ref)
1–4	135	112.3	2.66 [−2.29, 7.62]	−1.82 [−6.34, 2.70]	−1.59 [−6.05, 2.88]
None	407	108.3	−1.33 [−4.81, 2.15]	−3.63 [−6.70, −0.55]	−3.42 [−6.53, −0.31]
Clinical					Adjusted regression coefficient (95% CI)^b^,^d^
Body Mass Index (BMI) category (kg/m^2^)			*P* < 0.001	*P* = 0.08	*P* = 0.12
Underweight (<18.5)	109	108.0	−3.80 [−10.67, 3.06]	0.78 [−4.82, 6.38]	0.89 [−4.67, 6.46]
Normal (18.5–<25)	737	111.8	0 (ref)	0 (ref)	0 (ref)
Overweight (25–<30)	123	102.8	−8.94 [−13.98, −3.90]	−3.75 [−8.69, 1.19]	−3.67 [−8.68, 1.34]
Obese (≥30)	69	98.8	−13.00 [−17.34, −8.66]	−4.69 [−8.70, −0.69]	−4.32 [−8.34, −0.29]
Central Obesity			*P* = 0.02	*P* = 0.27	*P* = 0.34
No	784	111.4	0 (ref)	0 (ref)	0 (ref)
Yes	259	103.7	−7.70 [−11.77, −3.63]	−2.30 [−6.46, 1.87]	−1.97 [−6.07, 2.13]
Hypertension			*P* < 0.001	*P* = 0.03	*P* = 0.05
No	863	112.2	0 (ref)	0 (ref)	0 (ref)
Yes	180	96.2	−16.00 [−20.52, −11.49]	−5.45 [−10.44, −0.46]	−4.85 [−9.62, −0.09]
Systolic blood pressure					
<120 mmHg	448	114.4	*P* < 0.001 −3.72 [−4.68, −2.76]^g^	*P* = 0.006 −1.52 [–2.58, −0.46]^g^	*P* = 0.007 −1.48 [−2.52, −0.43]^g^
120–140 mmHg	445	109.2			
>140 mmHg	150	95.4			
Diastolic blood pressure					
<80 mmHg	748	112.7	*P* < 0.001 −5.71 [−7.42, −4.01]^g^	*P* = 0.03 −1.93 [−3.68, −0.17]^g^	*P* = 0.04 −1.87 [–3.62, −0.11]^g^
80–90 mmHg	210	102.7			
>90 mmHg	85	97.8			
Diabetes^e^			*P* = 0.49	*P* = 0.41	*P* = 0.32
No	1031	109.6	0 (ref)	0 (ref)	0 (ref)
Yes	9	102.9	−6.66 [−26.03, 12.71]	6.65 [−9.33, 22.62]	7.68 [−7.75, 23.10]
HIV positive^f^			*P* = 0.30	*P* = 0.40	*P* = 0.31
No	947	109.6	0 (ref)	0 (ref)	0 (ref)
Yes	87	106.8	−2.73 [−8.03, 2.57]	1.78 [−2.41, 5.96]	2.09 [−2.00, 6.17]

a Actual number of respondents and means, without sampling weights applied.

b Standard errors adjusted for clustering in survey design.

c Behavioural factors adjusted for age, sex, location and all independent sociodemographic predictors of GFR: number of items owned.

d Clinical factors adjusted for age, sex, location, number of items owned and independent behavioural predictors of GFR: days of vigorous physical activity/week.

e Missing for three participants.

f HIV diagnosis missing for nine participants.

g Regression coefficient for 10 unit mmHg increase in blood pressure modelled as a linear association with continuous covariate.

After adjusting for sociodemographic factors, there was evidence of a decrease in mean eGFR among those with no daily vigorous activity (−3.4 [−6.5, −0.3]) compared with those with who engaged in vigorous activity ≥5 days a week. There was no evidence of an association with other behavioural factors. We also observed a decrease in mean eGFR associated with increasing blood pressure (adjusted regression coefficients for each 10 mmHg increase in systolic or diastolic blood pressure: −1.5 [−2.5, −0.4] and −1.9 [−3.6, −0.1], respectively). There was also weak evidence of an association with BMI, with mean eGFR being lowest among obese participants (adjusted regression coefficient −4.3 [−8.3, −0.3]).

### Association of decreased renal function with other chronic diseases

The adjusted population attributable fraction (PAF) of decreased renal function due to HIV, hypertension and diabetes was 6%, 15% and <0.5%, respectively. The adjusted joint PAF of decreased renal function for all three risk factors was 21%. Supplementary Table 1 displays the association between decreased renal function and three major chronic diseases (hypertension, diabetes mellitus and HIV) in Mwanza city, district towns and rural areas. Of note, in district towns, only 7 of 23 adults with decreased renal function had any of these three chronic diseases.

## Discussion

Decreased renal function (eGFR<60 ml/min/1.73 m^2^) is common in north‐western Tanzania, particularly after taking into account the younger age this population. In our study, the age‐standardised prevalence of decreased renal function was higher in rural areas and district towns than the prevalence of decreased renal function reported from high‐income countries. Prevalences ranged from 9% in rural areas to 11% in district towns compared with 6% in the most recent national survey in the United States 15. To the best of our knowledge, this is the first population survey to describe and compare eGFR in adults in urban, semi‐urban and rural areas in SSA. Our results confirm and extend the results of previous study. In one recently published community survey of mainly urban adults in East Africa, the prevalence of kidney disease was 7% 16. Two population surveys of urban areas of Central and Southern Africa found a prevalence of eGFR<60 ml/min/1.73 m^2^ of 7–8% 17, 18, while in rural West Africa, the prevalence was 5% 10. These results are important because an eGFR of 30–60 ml/min/1.73 m^2^ has been associated with a 20–50% increased risk of all‐cause mortality and a 50–110% increased risk of cardiovascular mortality 19. The health risks related to decreased renal function may be even higher in African populations due to limited resources for the diagnosis and treatment of kidney disease 20, 21.

We described the factors associated with decreased renal function both as a binary and as a continuous variable. Findings in the two models supported one another. As expected, the factor most strongly associated with low eGFR was older age. Strikingly, though, decreased renal function was also common in younger age groups in our study. Among study subjects, 20‐ to 39‐year‐old dwelling in rural areas and district towns, 6–8% had eGFR < 60 ml/min/1.73 m^2^ compared to 0.7% in the US population 22.

Other factors significantly associated with lower eGFR included greater relative wealth (as determined by number of items owned) and high blood pressure. High blood pressure has consistently been shown to be been associated with decreased eGFR in prior studies from SSA 16, 17, 18. Due to the lower prevalence of diabetes mellitus and obesity (compared to developed countries), hypertension is relatively more important than these other factors as a risk factor for kidney disease in our region 5. Of note, diabetes mellitus was not associated with decreased renal function but this may have been due to both the low prevalence of diabetes mellitus and the fact that, in this community survey, we may have identified study subjects who had not had diabetes long enough to develop nephropathy. Decreased renal function was also associated with great wealth, as demonstrated in another study from East Africa 16, but not with higher income.

In our study, only 21% of the decreased renal function observed was attributable to the combination of hypertension (15%), HIV (6%) and/or diabetes mellitus (<0.5%). In contrast, a recent study reported that, worldwide, nearly 60% of kidney disease is jointly attributable to hypertension (~45%), diabetes mellitus (~25%) and/or high BMI (~25%) 23. Given that nearly 80% of decreased renal function observed in our study was unexplained by other chronic diseases, we are planning further studies to investigate the etiologies of kidney disease in our population. Schistosomiasis could be an important cause of kidney disease in our region 24, and we have already demonstrated a strong association between kidney disease and schistosomiasis in Tanzanian children 25. Post‐infectious glomerulonephritis is also thought to be one of the leading causes of kidney disease among adults in SSA 5, although data are lacking. Traditional herbal medicine use is also common in SSA, and some traditional herbs are nephrotoxic 26. Genetic factors such as apolipoprotein L1 (APOL1) variants may also contribute 27. These infectious, post‐infectious and genetic factors could also explain the comparatively high prevalence of decreased renal function that we observed in young adults. Further studies are needed to determine the relative contribution of these factors to renal disease in our region as well as other regions of SSA.

District towns had the highest prevalence of decreased renal function, although the explanation remains uncertain. Of note, mining is the major industry in the two district towns included in our study (Kahama and Geita). Small scale, artisanal gold miners in Tanzania use mercury, which easily enters into the soil and water supply 28 and can cause decreased renal function 29. Differences in incidence of glomerulonephritis or infections such as malaria could also be contributing. Kidney disease of unknown cause has also been observed in other poor, rural and agrarian regions around the globe and has been variously attributed to pesticide exposure, diet and hard manual labour 30. Differences in eGFR between district towns, cities and rural areas could also be explained by differences in muscle mass or protein intake. Further studies are needed to investigate these possible explanations.

Strengths of our study are its large size and its design as a population‐based representative household survey. Another strength is the use of the WHO STEPS questionnaire that allows comparison to other studies 8. In addition, our study was conducted in close collaboration with representatives of the Tanzanian Ministry of Health. Our results are helping to guide the Nephrology Society of Tanzania (NESOT), a partner of the Ministry of Health that was established in 2012, in their efforts to expand screening and treatment services for kidney disease in Tanzania. The NESOT community screening programme, which began in urban areas on World Kidney Day 2013, is now moving towards district towns and rural areas.

Our study also has limitations. First, because urine was not collected as part of our community survey, we could not measure either albuminuria or haematuria. As the number of participants with low eGFR was relatively small, particularly in urban areas, the resulting imprecision and wide confidence intervals of our estimates for urban areas may partly explain the low prevalence of decreased renal function that we observed. Furthermore, because there were few outcomes, our estimated ORs and 95% CIs for some risk factors may be biased. Lastly, the 72% response rate observed in our study might have led to non‐response bias, although 90% of households randomly selected for this study and 80% of individuals in those households did consent to participate. Survivor bias might also be present and could explain the low prevalence of advanced renal disease, as adults with advanced renal disease usually do not survive long in Tanzania.

In conclusion, our community‐based study of a random sample of urban and rural Tanzanian adults demonstrated a high prevalence of eGFR < 60 ml/min/1.73 m^2^ in district towns and rural areas. Low eGFR was more common in our population than in the US population, particularly in younger age groups. Only 20% of decreased renal function could be explained by hypertension, HIV or diabetes mellitus. Therefore, further research is needed to identify additional risk factors for kidney disease in SSA. Importantly, as in Tanzania, kidney disease seems to be even more common than many other chronic diseases (such as diabetes mellitus), health services for kidney disease require urgent attention. The best strategy for whom to screen is yet to be determined, but these data provide a starting point for planning clinical trials and health service improvements.

## Supporting information


**Table S1.** Overlap between decreased renal function and three major risk factors (hypertension, diabetes mellitus and HIV) stratified by location among adults in a population survey in northwestern Tanzania, 2012–2013.

## References

[1] Stanifer JW , Jing B , Tolan S *et al* The epidemiology of chronic kidney disease in sub‐Saharan Africa: a systematic review and meta‐analysis. Lancet Glob Heal 2014: 14: 1–8. 10.1016/S2214-109X(14)70002-6 25102850

[2] Peck RN , Green E , Mtabaji J *et al* Hypertension‐related diseases as a common cause of hospital mortality in Tanzania: a 3‐year prospective study. J Hypertens 2013: 31: 1806–1811. 23777761 10.1097/HJH.0b013e328362bad7 PMC4005815

[3] Luyckx VA , Naicker S , McKee M . Equity and economics of kidney disease in sub‐Saharan Africa. The Lancet 2013: 382: 103–104. 10.1016/S0140-6736(13)60817-X 23727172

[4] Garcia‐Garcia G , Jha V . CKD in disadvantaged populations. Kidney Int 2015: 87: 251–253. 25635713 10.1038/ki.2014.369

[5] Naicker S . End‐stage renal disease in Sub‐Saharan Africa. Kidney Int Suppl 2013: 3: 161–163. 10.1046/j.1523-1755.63.s83.25.x 12864889

[6] Dalal S , Beunza JJ , Volmink J *et al* Non‐communicable diseases in sub‐Saharan Africa: what we know now. Int J Epidemiol 2011: 40: 885–901. 21527446 10.1093/ije/dyr050

[7] Kavishe B , Biraro S , Baisley K *et al* High prevalence of hypertension and of risk factors for non‐communicable diseases (NCDs): a population based cross‐sectional survey of NCDS and HIV infection in Northwestern Tanzania and Southern Uganda. BMC Med 2015: 13: 126. 26021319 10.1186/s12916-015-0357-9 PMC4476208

[8] World Health Organization . WHO STEPS Instrument. World Health: Geneva, Switzerland, 2012 (Available from: http://www.who.int/chp/steps/instrument/en/) [13 Aug 2015].

[9] Kidney Disease Improving Global Outcomes . KDIGO 2012 clinical practice guideline for the evaluation and management of Chronic Kidney Disease. Kidney Int 2013: 3: 63–72. 10.1038/ki.2013.243 23989362

[10] Eastwood JB , Kerry SM , Plange‐Rhule J *et al* Assessment of GFR by four methods in adults in Ashanti, Ghana: the need for an eGFR equation for lean African populations. Nephrol Dial Transplant 2010: 25: 2178–2187. 20100724 10.1093/ndt/gfp765 PMC2891745

[11] Wyatt CM , Schwartz GJ , Owino Ong'or W *et al* Estimating kidney function in HIV‐infected adults in Kenya: comparison to a direct measure of glomerular filtration rate by iohexol clearance. PLoS One 2013: 8: e69601. 23950899 10.1371/journal.pone.0069601 PMC3738577

[12] Van Deventer HE , George JA , Paiker JE , Becker PJ , Katz IJ . Estimating glomerular filtration rate in black South Africans by use of the modification of diet in renal disease and Cockcroft‐Gault equations. Clin Chem 2008: 54: 1197–1202. 18487286 10.1373/clinchem.2007.099085

[13] Chobanian AV , Bakris GL , Black HR *et al* Seventh report of the joint national committee on prevention, detection, evaluation, and treatment of high blood pressure. Hypertension 2003: 42: 1–104. 14656957 10.1161/01.HYP.0000107251.49515.c2

[14] Landis JR , Koch GG . An application of hierarchical kappa‐type statistics in the assessment of majority agreement among multiple observers. Biometrics 1977: 33: 363–374. 884196

[15] National Health and Nutrition Examination Survey . Chronic kidney disease in the general population, 2013 (Available from: http://www.usrds.org/2012/pdf/v1_ch1_12.pdf) [13 Aug 2015].

[16] Stanifer JW , Maro V , Egger J *et al* The epidemiology of chronic kidney disease in Northern Tanzania: a population‐based survey. PLoS One 2015: 10: e0124506. 25886472 10.1371/journal.pone.0124506 PMC4401757

[17] Matsha TE , Yako YY , Rensburg MA , Hassan MS , Kengne AP & Erasmus RT . Chronic kidney diseases in mixed ancestry south African populations: prevalence, determinants and concordance between kidney function estimators. BMC Nephrol 2013: 14: 75. 23547953 10.1186/1471-2369-14-75 PMC3637389

[18] Sumaili EK , Krzesinski J‐M , Zinga CV *et al* Prevalence of chronic kidney disease in Kinshasa: results of a pilot study from the Democratic Republic of Congo. Nephrol Dial Transplant 2009: 24: 117–122. 18715963 10.1093/ndt/gfn469

[19] Fox CS , Matsushita K , Woodward M *et al* Associations of kidney disease measures with mortality and end‐stage renal disease in individuals with and without diabetes: a meta‐analysis. Lancet 2012: 380: 1662–1673. 23013602 10.1016/S0140-6736(12)61350-6 PMC3771350

[20] Peck R , Mghamba J , Vanobberghen F *et al* Preparedness of Tanzanian health facilities for outpatient primary care of hypertension and diabetes: a cross‐sectional survey. Lancet Glob Heal 2014: 14: 1–8. 10.1016/S2214-109X(14)70033-6 PMC4013553 24818084

[21] Kilonzo K , Mathew A , Croome AJ . Establishment of an acute peritoneal dialysis program in Tanzania. Kidney Int Suppl 2013: 3: 186–189.

[22] Coresh J , Selvin E , Stevens LA *et al* Prevalence of chronic kidney disease in the United States. JAMA 2007: 298: 2038–2047. 17986697 10.1001/jama.298.17.2038

[23] The Global Burden of Metabolic Risk Factors for Chronic Diseases Collaboration . Cardiovascular disease, chronic kidney disease, and diabetes mortality burden of cardiometabolic risk factors from 1980 to 2010: a comparative risk assessment. Lancet Diabetes Endocrinol 2014: 8587: 1–14. 10.1016/S2213-8587(14)70102-0 PMC4572741 24842598

[24] Barsoum R . The changing face of schistosomal glomerulopathy. Kidney Int 2004: 66: 2472–2484. 15569345 10.1111/j.1523-1755.2004.66042.x

[25] Kayange NM , Smart LR , Downs JA , Maskini M , Fitzgerald DW , Peck RN . The influence of HIV and Schistosomiasis on renal function: a cross‐sectional study among children at a hospital in Tanzania. PLoS Negl Trop Dis 2015: 9: e0003472. 25612312 10.1371/journal.pntd.0003472 PMC4303314

[26] Liwa AC , Smart LR , Frumkin A , Epstein H‐AB , Fitzgerald DW , Peck RN . Traditional herbal medicine use among hypertensive patients in sub‐saharan Africa: a systematic review. Curr Hypertens Rep 2014: 16: 437. 24764197 10.1007/s11906-014-0437-9 PMC4076776

[27] Parsa A , Kao WHL , Xie D *et al* APOL1 risk variants, race, and progression of chronic kidney disease. N Engl J Med 2013: 369: 2183–2196. 24206458 10.1056/NEJMoa1310345 PMC3969022

[28] Nyanza EC , Dewey D , Thomas DSK , Davey M , Ngallaba SE . Spatial distribution of mercury and arsenic levels in water, soil and cassava plants in a community with long history of gold mining in Tanzania. Bull Environ Contam Toxicol 2014: 93: 716–721. 24923470 10.1007/s00128-014-1315-5

[29] Barbier O , Jacquillet G , Tauc M , Cougnon M , Poujeol P . Effect of heavy metals on, and handling by, the kidney. Nephron Physiol 2005: 99: 105–110. 10.1159/000083981 15722646

[30] Yaqub F . Kidney disease in farming communities remains a mystery. Lancet 2014: 383: 1794–1795. 24868568 10.1016/s0140-6736(14)60867-9

