# Supplementary material for: Decreased renal function and associated factors in cities, towns and rural areas of Tanzania: a community‐based population survey
Source: Trop Med Int Health. 2015 Dec 28;21(3):393–404. doi: 10.1111/tmi.12651 (PMC4784164; doi:10.1111/tmi.12651)
Supplement: Supplementary file 1 — Table S1. Overlap between decreased renal function and three major risk factors (hypertension, diabetes mellitus and HIV) stratified by location among adults in a population survey in northwestern Tanzania, 2012–2013. [file TMI-21-393-s001.docx]

Supplementary Table 1: Overlap between decreased renal function and 3 major risk factors (hypertension, diabetes mellitus and HIV) stratified by location among adults in a population survey in northwestern Tanzania, 2012-2013

|  | **Mwanza city** | | **District towns** | | **Rural** | |
| --- | --- | --- | --- | --- | --- | --- |
|  | Prevalence  (95% CI) ^1^ | N^2^ | Prevalence  (95% CI) ^1^ | N^2^ | Prevalence  (95% CI) ^1^ | N^2^ |
| ***Among those with kidney disease*** | | | | | | |
| ***Total*** |  | ***4*** |  | ***23*** |  | ***42*** |
| Hypertension | 53.6% (5.3-96.0) | 2 | 17.4% (7.2-36.5) | 4 | 40.6% (23.5-60.3) | 17 |
| Diabetes | 0 | 0 | 0 | 0 | 3.0 % (0.4-19.7) | 1 |
| HIV positive | 23.2% (3.5-71.5) | 1 | 17.2% (6.5-38.4) | 4 | 9.0 % (3.9-19.2) | 4 |
| Any of the above | 76.8% (28.5-96.5) | 3 | 29.4% (13.4-52.7) | 7 | 46.2% (29.7-63.6) | 20 |
| ***Among those with hypertension*** | | | | | | |
| ***Total*** |  | ***27*** |  | ***52*** |  | ***101*** |
| Kidney disease | 7.9% (1.4-34.6) | 2 | 8.0% (2.8-21.2) | 4 | 17.3% (2.9-24.2) | 17 |
| ***Among those with diabetes*** | | | | | | |
| ***Total*** |  | ***2*** |  | ***4*** |  | ***3*** |
| Kidney disease | 0 | 0 | 0 | 0 | 37.6% (16.3-65.2) | 1 |
| ***Among those with HIV*** | | | | | | |
| ***Total*** |  | ***15*** |  | ***35*** |  | ***37*** |
| Kidney disease | 6.0% (0.9-30.0) | 1 | 11.8% (4.5-27.7) | 4 | 9.9% (5.0-18.7) | 4 |

^1^Prevalence and 95% CI are weighted estimates, adjusted for survey design with sampling weights applied. **^2^**Actual number of respondents, without sampling weights applied
